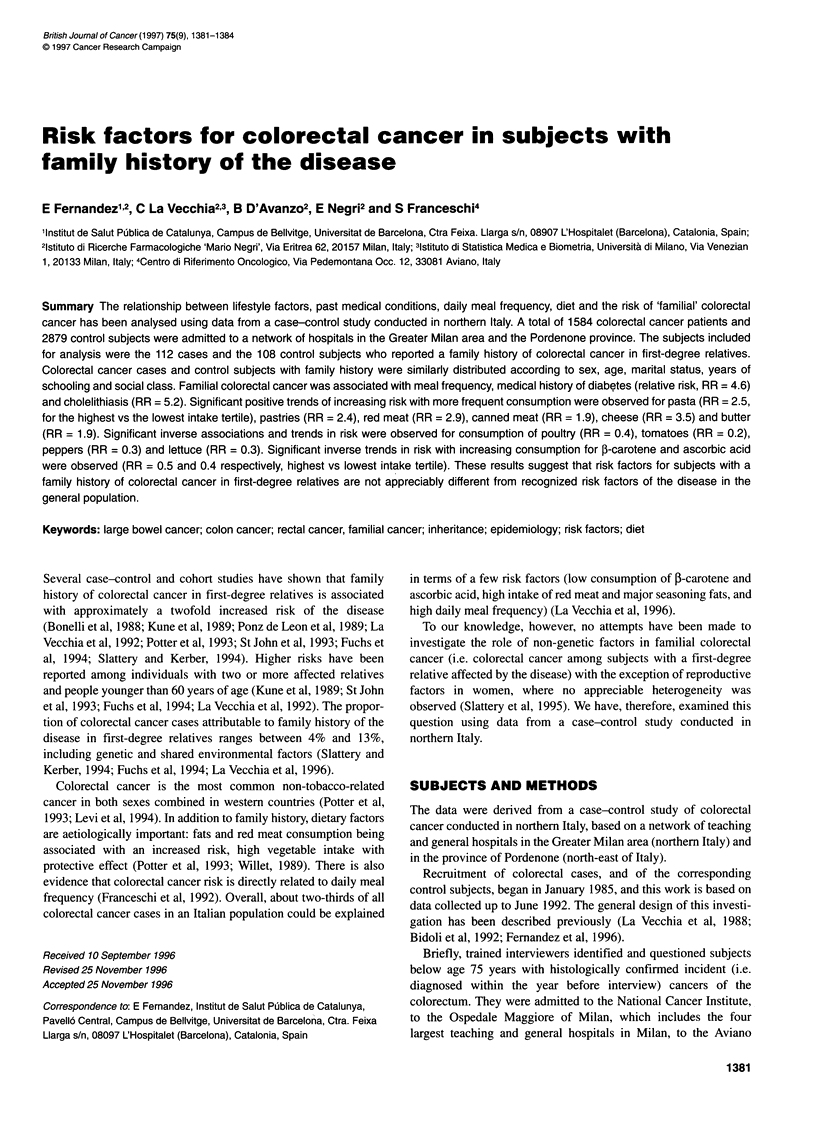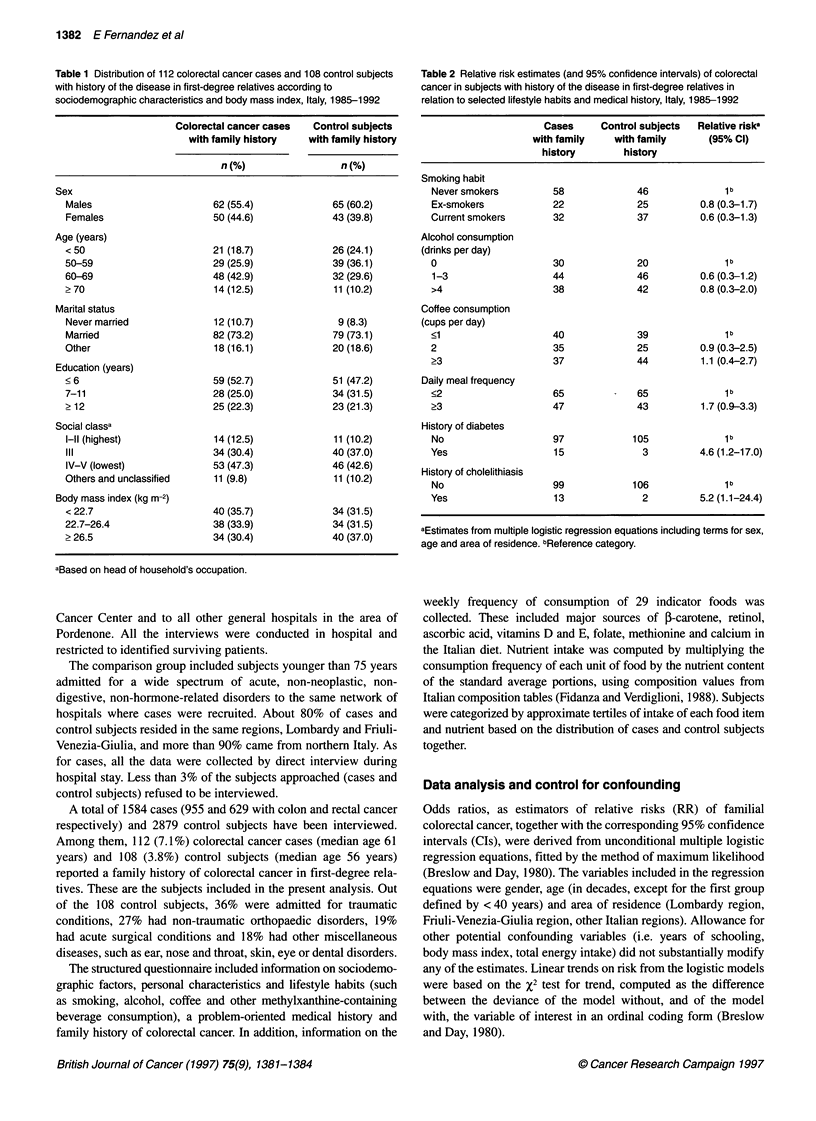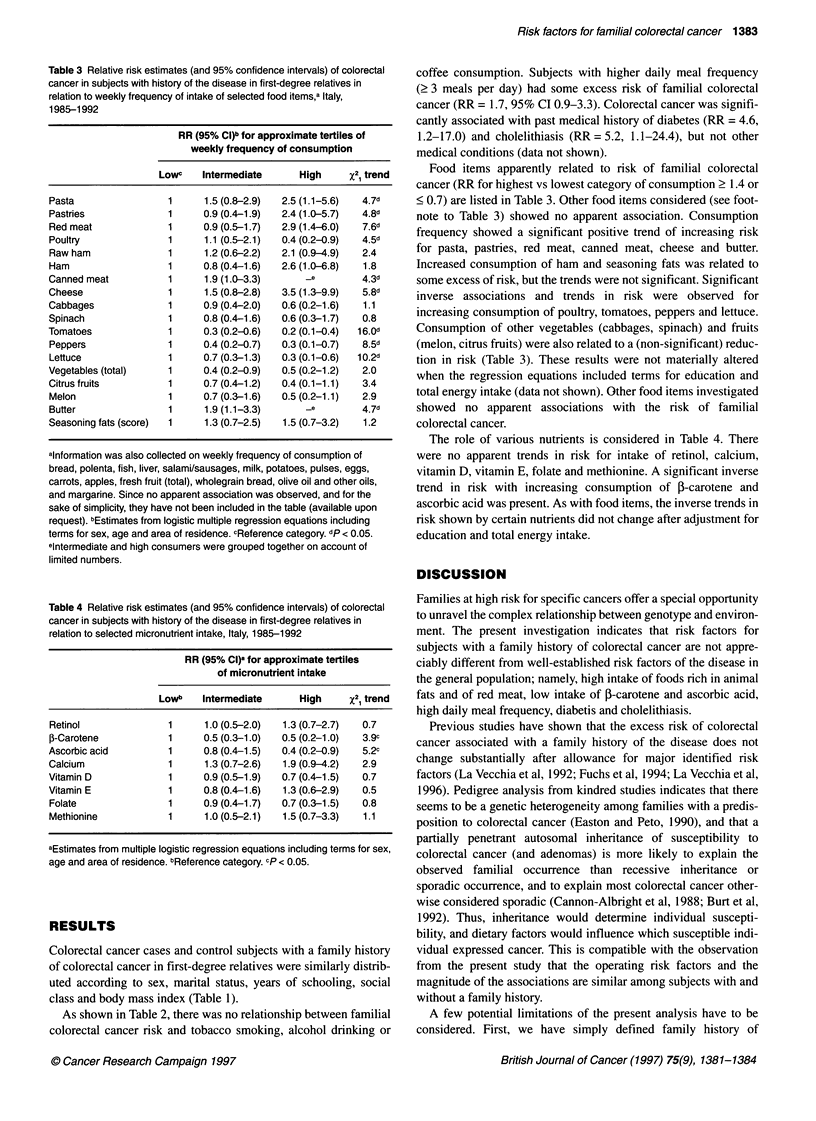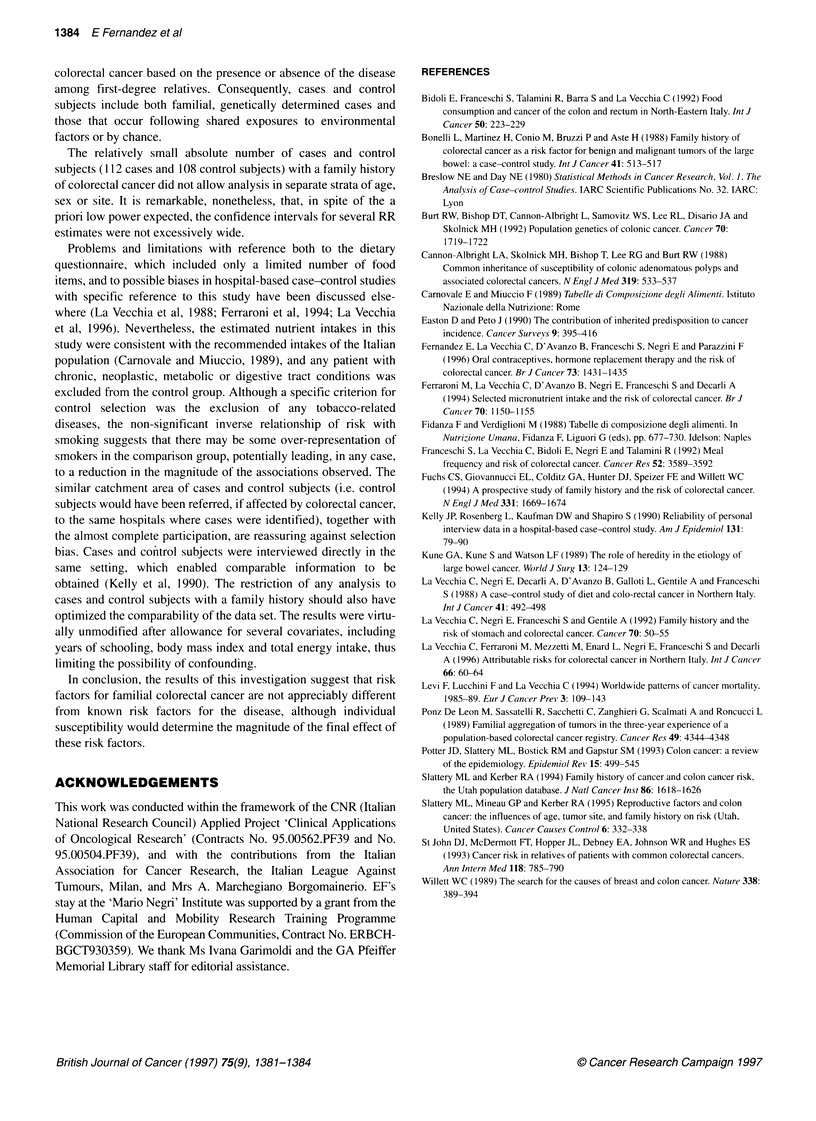# Risk factors for colorectal cancer in subjects with family history of the disease.

**DOI:** 10.1038/bjc.1997.234

**Published:** 1997

**Authors:** E. Fernandez, C. La Vecchia, B. D'Avanzo, E. Negri, S. Franceschi

**Affiliations:** Institut de Salut PÃºblica de Catalunya, Campus de Bellvitge, Universitat de Barcelona, L'Hospitalet, Catalonia, Spain.

## Abstract

The relationship between lifestyle factors, past medical conditions, daily meal frequency, diet and the risk of 'familial' colorectal cancer has been analysed using data from a case-control study conducted in northern Italy. A total of 1584 colorectal cancer patients and 2879 control subjects were admitted to a network of hospitals in the Greater Milan area and the Pordenone province. The subjects included for analysis were the 112 cases and the 108 control subjects who reported a family history of colorectal cancer in first-degree relatives. Colorectal cancer cases and control subjects with family history were similarly distributed according to sex, age, marital status, years of schooling and social class. Familial colorectal cancer was associated with meal frequency, medical history of diabetes (relative risk, RR = 4.6) and cholelithiasis (RR = 5.2). Significant positive trends of increasing risk with more frequent consumption were observed for pasta (RR = 2.5, for the highest vs the lowest intake tertile), pastries (RR = 2.4), red meat (RR = 2.9), canned meat (RR = 1.9), cheese (RR = 3.5) and butter (RR = 1.9). Significant inverse associations and trends in risk were observed for consumption of poultry (RR = 0.4), tomatoes (RR = 0.2), peppers (RR = 0.3) and lettuce (RR = 0.3). Significant inverse trends in risk with increasing consumption for beta-carotene and ascorbic acid were observed (RR = 0.5 and 0.4 respectively, highest vs lowest intake tertile). These results suggest that risk factors for subjects with a family history of colorectal cancer in first-degree relatives are not appreciably different from recognized risk factors of the disease in the general population.


British Journal of Cancer (1997) 75(9), 1381–1384
© 1997 Cancer Research Campaign

### Risk factors for colorectal cancer in subjects with family history of the disease

**E Fernandez[1,2], C La Vecchia[2,3], B D'Avanzo[2], E Negri[2] and S Franceschi[4]**

[1]Institut de Salut Pública de Catalunya, Campus de Bellvitge, Universitat de Barcelona, Ctra Feixa. Llarga s/n, 08907 L'Hospitalet (Barcelona), Catalonia, Spain; [2]Istituto di Ricerche Farmacologiche 'Mario Negri', Via Eritrea 62, 20157 Milan, Italy; [3]Istituto di Statistica Medica e Biometria, Università di Milano, Via Venezian 1, 20133 Milan, Italy; [4]Centro di Riferimento Oncologico, Via Pedemontana Occ. 12, 33081 Aviano, Italy

**Summary** The relationship between lifestyle factors, past medical conditions, daily meal frequency, diet and the risk of 'familial' colorectal cancer has been analysed using data from a case–control study conducted in northern Italy. A total of 1584 colorectal cancer patients and 2879 control subjects were admitted to a network of hospitals in the Greater Milan area and the Pordenone province. The subjects included for analysis were the 112 cases and the 108 control subjects who reported a family history of colorectal cancer in first-degree relatives. Colorectal cancer cases and control subjects with family history were similarly distributed according to sex, age, marital status, years of schooling and social class. Familial colorectal cancer was associated with meal frequency, medical history of diabetes (relative risk, RR = 4.6) and cholelithiasis (RR = 5.2). Significant positive trends of increasing risk with more frequent consumption were observed for pasta (RR = 2.5, for the highest vs the lowest intake tertile), pastries (RR = 2.4), red meat (RR = 2.9), canned meat (RR = 1.9), cheese (RR = 3.5) and butter (RR = 1.9). Significant inverse associations and trends in risk were observed for consumption of poultry (RR = 0.4), tomatoes (RR = 0.2), peppers (RR = 0.3) and lettuce (RR = 0.3). Significant inverse trends in risk with increasing consumption for β-carotene and ascorbic acid were observed (RR = 0.5 and 0.4 respectively, highest vs lowest intake tertile). These results suggest that risk factors for subjects with a family history of colorectal cancer in first-degree relatives are not appreciably different from recognized risk factors of the disease in the general population.

**Keywords:** large bowel cancer; colon cancer; rectal cancer, familial cancer; inheritance; epidemiology; risk factors; diet

Several case–control and cohort studies have shown that family history of colorectal cancer in first-degree relatives is associated with approximately a twofold increased risk of the disease (Bonelli et al, 1988; Kune et al, 1989; Ponz de Leon et al, 1989; La Vecchia et al, 1992; Potter et al, 1993; St John et al, 1993; Fuchs et al, 1994; Slattery and Kerber, 1994). Higher risks have been reported among individuals with two or more affected relatives and people younger than 60 years of age (Kune et al, 1989; St John et al, 1993; Fuchs et al, 1994; La Vecchia et al, 1992). The proportion of colorectal cancer cases attributable to family history of the disease in first-degree relatives ranges between 4% and 13%, including genetic and shared environmental factors (Slattery and Kerber, 1994; Fuchs et al, 1994; La Vecchia et al, 1996).

Colorectal cancer is the most common non-tobacco-related cancer in both sexes combined in western countries (Potter et al, 1993; Levi et al, 1994). In addition to family history, dietary factors are aetiologically important: fats and red meat consumption being associated with an increased risk, high vegetable intake with protective effect (Potter et al, 1993; Willet, 1989). There is also evidence that colorectal cancer risk is directly related to daily meal frequency (Franceschi et al, 1992). Overall, about two-thirds of all colorectal cancer cases in an Italian population could be explained in terms of a few risk factors (low consumption of β-carotene and ascorbic acid, high intake of red meat and major seasoning fats, and high daily meal frequency) (La Vecchia et al, 1996).

To our knowledge, however, no attempts have been made to investigate the role of non-genetic factors in familial colorectal cancer (i.e. colorectal cancer among subjects with a first-degree relative affected by the disease) with the exception of reproductive factors in women, where no appreciable heterogeneity was observed (Slattery et al, 1995). We have, therefore, examined this question using data from a case–control study conducted in northern Italy.

### SUBJECTS AND METHODS

The data were derived from a case–control study of colorectal cancer conducted in northern Italy, based on a network of teaching and general hospitals in the Greater Milan area (northern Italy) and in the province of Pordenone (north-east of Italy).

Recruitment of colorectal cases, and of the corresponding control subjects, began in January 1985, and this work is based on data collected up to June 1992. The general design of this investigation has been described previously (La Vecchia et al, 1988; Bidoli et al, 1992; Fernandez et al, 1996).

Briefly, trained interviewers identified and questioned subjects below age 75 years with histologically confirmed incident (i.e. diagnosed within the year before interview) cancers of the colorectum. They were admitted to the National Cancer Institute, to the Ospedale Maggiore of Milan, which includes the four largest teaching and general hospitals in Milan, to the Aviano

*Received 10 September 1996*
*Revised 25 November 1996*
*Accepted 25 November 1996*

*Correspondence to*: E Fernandez, Institut de Salut Pública de Catalunya, Pavelló Central, Campus de Bellvitge, Universitat de Barcelona, Ctra. Feixa Llarga s/n, 08097 L'Hospitalet (Barcelona), Catalonia, Spain

**Table 1** Distribution of 112 colorectal cancer cases and 108 control subjects with history of the disease in first-degree relatives according to sociodemographic characteristics and body mass index, Italy, 1985–1992

| | Colorectal cancer cases with family history | Control subjects with family history |
|---|---|---|
| | n (%) | n (%) |
| Sex | | |
| Males | 62 (55.4) | 65 (60.2) |
| Females | 50 (44.6) | 43 (39.8) |
| Age (years) | | |
| < 50 | 21 (18.7) | 26 (24.1) |
| 50–59 | 29 (25.9) | 39 (36.1) |
| 60–69 | 48 (42.9) | 32 (29.6) |
| ≥ 70 | 14 (12.5) | 11 (10.2) |
| Marital status | | |
| Never married | 12 (10.7) | 9 (8.3) |
| Married | 82 (73.2) | 79 (73.1) |
| Other | 18 (16.1) | 20 (18.6) |
| Education (years) | | |
| ≤ 6 | 59 (52.7) | 51 (47.2) |
| 7–11 | 28 (25.0) | 34 (31.5) |
| ≥ 12 | 25 (22.3) | 23 (21.3) |
| Social class[a] | | |
| I–II (highest) | 14 (12.5) | 11 (10.2) |
| III | 34 (30.4) | 40 (37.0) |
| IV–V (lowest) | 53 (47.3) | 46 (42.6) |
| Others and unclassified | 11 (9.8) | 11 (10.2) |
| Body mass index (kg m$^{-2}$) | | |
| < 22.7 | 40 (35.7) | 34 (31.5) |
| 22.7–26.4 | 38 (33.9) | 34 (31.5) |
| ≥ 26.5 | 34 (30.4) | 40 (37.0) |

[a]Based on head of household's occupation.

**Table 2** Relative risk estimates (and 95% confidence intervals) of colorectal cancer in subjects with history of the disease in first-degree relatives in relation to selected lifestyle habits and medical history, Italy, 1985–1992

| | Cases with family history | Control subjects with family history | Relative risk[a] (95% CI) |
|---|---|---|---|
| Smoking habit | | | |
| Never smokers | 58 | 46 | 1[b] |
| Ex-smokers | 22 | 25 | 0.8 (0.3–1.7) |
| Current smokers | 32 | 37 | 0.6 (0.3–1.3) |
| Alcohol consumption (drinks per day) | | | |
| 0 | 30 | 20 | 1[b] |
| 1–3 | 44 | 46 | 0.6 (0.3–1.2) |
| >4 | 38 | 42 | 0.8 (0.3–2.0) |
| Coffee consumption (cups per day) | | | |
| ≤1 | 40 | 39 | 1[b] |
| 2 | 35 | 25 | 0.9 (0.3–2.5) |
| ≥3 | 37 | 44 | 1.1 (0.4–2.7) |
| Daily meal frequency | | | |
| ≤2 | 65 | 65 | 1[b] |
| ≥3 | 47 | 43 | 1.7 (0.9–3.3) |
| History of diabetes | | | |
| No | 97 | 105 | 1[b] |
| Yes | 15 | 3 | 4.6 (1.2–17.0) |
| History of cholelithiasis | | | |
| No | 99 | 106 | 1[b] |
| Yes | 13 | 2 | 5.2 (1.1–24.4) |

[a]Estimates from multiple logistic regression equations including terms for sex, age and area of residence. [b]Reference category.

Cancer Center and to all other general hospitals in the area of Pordenone. All the interviews were conducted in hospital and restricted to identified surviving patients.

The comparison group included subjects younger than 75 years admitted for a wide spectrum of acute, non-neoplastic, non-digestive, non-hormone-related disorders to the same network of hospitals where cases were recruited. About 80% of cases and control subjects resided in the same regions, Lombardy and Friuli-Venezia-Giulia, and more than 90% came from northern Italy. As for cases, all the data were collected by direct interview during hospital stay. Less than 3% of the subjects approached (cases and control subjects) refused to be interviewed.

A total of 1584 cases (955 and 629 with colon and rectal cancer respectively) and 2879 control subjects have been interviewed. Among them, 112 (7.1%) colorectal cancer cases (median age 61 years) and 108 (3.8%) control subjects (median age 56 years) reported a family history of colorectal cancer in first-degree relatives. These are the subjects included in the present analysis. Out of the 108 control subjects, 36% were admitted for traumatic conditions, 27% had non-traumatic orthopaedic disorders, 19% had acute surgical conditions and 18% had other miscellaneous diseases, such as ear, nose and throat, skin, eye or dental disorders.

The structured questionnaire included information on sociodemographic factors, personal characteristics and lifestyle habits (such as smoking, alcohol, coffee and other methylxanthine-containing beverage consumption), a problem-oriented medical history and family history of colorectal cancer. In addition, information on the weekly frequency of consumption of 29 indicator foods was collected. These included major sources of β-carotene, retinol, ascorbic acid, vitamins D and E, folate, methionine and calcium in the Italian diet. Nutrient intake was computed by multiplying the consumption frequency of each unit of food by the nutrient content of the standard average portions, using composition values from Italian composition tables (Fidanza and Verdiglioni, 1988). Subjects were categorized by approximate tertiles of intake of each food item and nutrient based on the distribution of cases and control subjects together.

### Data analysis and control for confounding

Odds ratios, as estimators of relative risks (RR) of familial colorectal cancer, together with the corresponding 95% confidence intervals (CIs), were derived from unconditional multiple logistic regression equations, fitted by the method of maximum likelihood (Breslow and Day, 1980). The variables included in the regression equations were gender, age (in decades, except for the first group defined by < 40 years) and area of residence (Lombardy region, Friuli-Venezia-Giulia region, other Italian regions). Allowance for other potential confounding variables (i.e. years of schooling, body mass index, total energy intake) did not substantially modify any of the estimates. Linear trends on risk from the logistic models were based on the $\chi^2$ test for trend, computed as the difference between the deviance of the model without, and of the model with, the variable of interest in an ordinal coding form (Breslow and Day, 1980).

 © Cancer Research Campaign 1997

**Table 3** Relative risk estimates (and 95% confidence intervals) of colorectal cancer in subjects with history of the disease in first-degree relatives in relation to weekly frequency of intake of selected food items,[a] Italy, 1985–1992

| | RR (95% CI)[b] for approximate tertiles of weekly frequency of consumption | | | |
|---|---|---|---|---|
| | Low[c] | Intermediate | High | $\chi^2_1$ trend |
| Pasta | 1 | 1.5 (0.8–2.9) | 2.5 (1.1–5.6) | 4.7[d] |
| Pastries | 1 | 0.9 (0.4–1.9) | 2.4 (1.0–5.7) | 4.8[d] |
| Red meat | 1 | 0.9 (0.5–1.7) | 2.9 (1.4–6.0) | 7.6[d] |
| Poultry | 1 | 1.1 (0.5–2.1) | 0.4 (0.2–0.9) | 4.5[d] |
| Raw ham | 1 | 1.2 (0.6–2.2) | 2.1 (0.9–4.9) | 2.4 |
| Ham | 1 | 0.8 (0.4–1.6) | 2.6 (1.0–6.8) | 1.8 |
| Canned meat | 1 | 1.9 (1.0–3.3) | –[e] | 4.3[d] |
| Cheese | 1 | 1.5 (0.8–2.8) | 3.5 (1.3–9.9) | 5.8[d] |
| Cabbages | 1 | 0.9 (0.4–2.0) | 0.6 (0.2–1.6) | 1.1 |
| Spinach | 1 | 0.8 (0.4–1.6) | 0.6 (0.3–1.7) | 0.8 |
| Tomatoes | 1 | 0.3 (0.2–0.6) | 0.2 (0.1–0.4) | 16.0[d] |
| Peppers | 1 | 0.4 (0.2–0.7) | 0.3 (0.1–0.7) | 8.5[d] |
| Lettuce | 1 | 0.7 (0.3–1.3) | 0.3 (0.1–0.6) | 10.2[d] |
| Vegetables (total) | 1 | 0.4 (0.2–0.9) | 0.5 (0.2–1.2) | 2.0 |
| Citrus fruits | 1 | 0.7 (0.4–1.2) | 0.4 (0.1–1.1) | 3.4 |
| Melon | 1 | 0.7 (0.3–1.6) | 0.5 (0.2–1.1) | 2.9 |
| Butter | 1 | 1.9 (1.1–3.3) | –[e] | 4.7[d] |
| Seasoning fats (score) | 1 | 1.3 (0.7–2.5) | 1.5 (0.7–3.2) | 1.2 |

[a]Information was also collected on weekly frequency of consumption of bread, polenta, fish, liver, salami/sausages, milk, potatoes, pulses, eggs, carrots, apples, fresh fruit (total), wholegrain bread, olive oil and other oils, and margarine. Since no apparent association was observed, and for the sake of simplicity, they have not been included in the table (available upon request). [b]Estimates from logistic multiple regression equations including terms for sex, age and area of residence. [c]Reference category. [d]$P < 0.05$. [e]Intermediate and high consumers were grouped together on account of limited numbers.

**Table 4** Relative risk estimates (and 95% confidence intervals) of colorectal cancer in subjects with history of the disease in first-degree relatives in relation to selected micronutrient intake, Italy, 1985–1992

| | RR (95% CI)[a] for approximate tertiles of micronutrient intake | | | |
|---|---|---|---|---|
| | Low[b] | Intermediate | High | $\chi^2_1$ trend |
| Retinol | 1 | 1.0 (0.5–2.0) | 1.3 (0.7–2.7) | 0.7 |
| β-Carotene | 1 | 0.5 (0.3–1.0) | 0.5 (0.2–1.0) | 3.9[c] |
| Ascorbic acid | 1 | 0.8 (0.4–1.5) | 0.4 (0.2–0.9) | 5.2[c] |
| Calcium | 1 | 1.3 (0.7–2.6) | 1.9 (0.9–4.2) | 2.9 |
| Vitamin D | 1 | 0.9 (0.5–1.9) | 0.7 (0.4–1.5) | 0.7 |
| Vitamin E | 1 | 0.8 (0.4–1.6) | 1.3 (0.6–2.9) | 0.5 |
| Folate | 1 | 0.9 (0.4–1.7) | 0.7 (0.3–1.5) | 0.8 |
| Methionine | 1 | 1.0 (0.5–2.1) | 1.5 (0.7–3.3) | 1.1 |

[a]Estimates from multiple logistic regression equations including terms for sex, age and area of residence. [b]Reference category. [c]$P < 0.05$.

### RESULTS

Colorectal cancer cases and control subjects with a family history of colorectal cancer in first-degree relatives were similarly distributed according to sex, marital status, years of schooling, social class and body mass index (Table 1).

As shown in Table 2, there was no relationship between familial colorectal cancer risk and tobacco smoking, alcohol drinking or coffee consumption. Subjects with higher daily meal frequency (≥ 3 meals per day) had some excess risk of familial colorectal cancer (RR = 1.7, 95% CI 0.9–3.3). Colorectal cancer was significantly associated with past medical history of diabetes (RR = 4.6, 1.2–17.0) and cholelithiasis (RR = 5.2, 1.1–24.4), but not other medical conditions (data not shown).

Food items apparently related to risk of familial colorectal cancer (RR for highest vs lowest category of consumption ≥ 1.4 or ≤ 0.7) are listed in Table 3. Other food items considered (see footnote to Table 3) showed no apparent association. Consumption frequency showed a significant positive trend of increasing risk for pasta, pastries, red meat, canned meat, cheese and butter. Increased consumption of ham and seasoning fats was related to some excess of risk, but the trends were not significant. Significant inverse associations and trends in risk were observed for increasing consumption of poultry, tomatoes, peppers and lettuce. Consumption of other vegetables (cabbages, spinach) and fruits (melon, citrus fruits) were also related to a (non-significant) reduction in risk (Table 3). These results were not materially altered when the regression equations included terms for education and total energy intake (data not shown). Other food items investigated showed no apparent associations with the risk of familial colorectal cancer.

The role of various nutrients is considered in Table 4. There were no apparent trends in risk for intake of retinol, calcium, vitamin D, vitamin E, folate and methionine. A significant inverse trend in risk with increasing consumption of β-carotene and ascorbic acid was present. As with food items, the inverse trends in risk shown by certain nutrients did not change after adjustment for education and total energy intake.

### DISCUSSION

Families at high risk for specific cancers offer a special opportunity to unravel the complex relationship between genotype and environment. The present investigation indicates that risk factors for subjects with a family history of colorectal cancer are not appreciably different from well-established risk factors of the disease in the general population; namely, high intake of foods rich in animal fats and of red meat, low intake of β-carotene and ascorbic acid, high daily meal frequency, diabetis and cholelithiasis.

Previous studies have shown that the excess risk of colorectal cancer associated with a family history of the disease does not change substantially after allowance for major identified risk factors (La Vecchia et al, 1992; Fuchs et al, 1994; La Vecchia et al, 1996). Pedigree analysis from kindred studies indicates that there seems to be a genetic heterogeneity among families with a predisposition to colorectal cancer (Easton and Peto, 1990), and that a partially penetrant autosomal inheritance of susceptibility to colorectal cancer (and adenomas) is more likely to explain the observed familial occurrence than recessive inheritance or sporadic occurrence, and to explain most colorectal cancer otherwise considered sporadic (Cannon-Albright et al, 1988; Burt et al, 1992). Thus, inheritance would determine individual susceptibility, and dietary factors would influence which susceptible individual expressed cancer. This is compatible with the observation from the present study that the operating risk factors and the magnitude of the associations are similar among subjects with and without a family history.

A few potential limitations of the present analysis have to be considered. First, we have simply defined family history of

© Cancer Research Campaign 1997

colorectal cancer based on the presence or absence of the disease among first-degree relatives. Consequently, cases and control subjects include both familial, genetically determined cases and those that occur following shared exposures to environmental factors or by chance.

The relatively small absolute number of cases and control subjects (112 cases and 108 control subjects) with a family history of colorectal cancer did not allow analysis in separate strata of age, sex or site. It is remarkable, nonetheless, that, in spite of the a priori low power expected, the confidence intervals for several RR estimates were not excessively wide.

Problems and limitations with reference both to the dietary questionnaire, which included only a limited number of food items, and to possible biases in hospital-based case–control studies with specific reference to this study have been discussed elsewhere (La Vecchia et al, 1988; Ferraroni et al, 1994; La Vecchia et al, 1996). Nevertheless, the estimated nutrient intakes in this study were consistent with the recommended intakes of the Italian population (Carnovale and Miuccio, 1989), and any patient with chronic, neoplastic, metabolic or digestive tract conditions was excluded from the control group. Although a specific criterion for control selection was the exclusion of any tobacco-related diseases, the non-significant inverse relationship of risk with smoking suggests that there may be some over-representation of smokers in the comparison group, potentially leading, in any case, to a reduction in the magnitude of the associations observed. The similar catchment area of cases and control subjects (i.e. control subjects would have been referred, if affected by colorectal cancer, to the same hospitals where cases were identified), together with the almost complete participation, are reassuring against selection bias. Cases and control subjects were interviewed directly in the same setting, which enabled comparable information to be obtained (Kelly et al, 1990). The restriction of any analysis to cases and control subjects with a family history should also have optimized the comparability of the data set. The results were virtually unmodified after allowance for several covariates, including years of schooling, body mass index and total energy intake, thus limiting the possibility of confounding.

In conclusion, the results of this investigation suggest that risk factors for familial colorectal cancer are not appreciably different from known risk factors for the disease, although individual susceptibility would determine the magnitude of the final effect of these risk factors.

### ACKNOWLEDGEMENTS

This work was conducted within the framework of the CNR (Italian National Research Council) Applied Project 'Clinical Applications of Oncological Research' (Contracts No. 95.00562.PF39 and No. 95.00504.PF39), and with the contributions from the Italian Association for Cancer Research, the Italian League Against Tumours, Milan, and Mrs A. Marchegiano Borgomainerio. EF's stay at the 'Mario Negri' Institute was supported by a grant from the Human Capital and Mobility Research Training Programme (Commission of the European Communities, Contract No. ERBCHBGCT930359). We thank Ms Ivana Garimoldi and the GA Pfeiffer Memorial Library staff for editorial assistance.

### REFERENCES

Bidoli E, Franceschi S, Talamini R, Barra S and La Vecchia C (1992) Food consumption and cancer of the colon and rectum in North-Eastern Italy. *Int J Cancer* **50**: 223–229

Bonelli L, Martinez H, Conio M, Bruzzi P and Aste H (1988) Family history of colorectal cancer as a risk factor for benign and malignant tumors of the large bowel: a case–control study. *Int J Cancer* **41**: 513–517

Breslow NE and Day NE (1980) *Statistical Methods in Cancer Research, Vol. 1. The Analysis of Case–control Studies*. IARC Scientific Publications No. 32. IARC: Lyon

Burt RW, Bishop DT, Cannon-Albright L, Samovitz WS, Lee RL, Disario JA and Skolnick MH (1992) Population genetics of colonic cancer. *Cancer* **70**: 1719–1722

Cannon-Albright LA, Skolnick MH, Bishop T, Lee RG and Burt RW (1988) Common inheritance of susceptibility of colonic adenomatous polyps and associated colorectal cancers. *N Engl J Med* **319**: 533–537

Carnovale E and Miuccio F (1989) *Tabelle di Composizione degli Alimenti*. Istituto Nazionale della Nutrizione: Rome

Easton D and Peto J (1990) The contribution of inherited predisposition to cancer incidence. *Cancer Surveys* **9**: 395–416

Fernandez E, La Vecchia C, D'Avanzo B, Franceschi S, Negri E and Parazzini F (1996) Oral contraceptives, hormone replacement therapy and the risk of colorectal cancer. *Br J Cancer* **73**: 1431–1435

Ferraroni M, La Vecchia C, D'Avanzo B, Negri E, Franceschi S and Decarli A (1994) Selected micronutrient intake and the risk of colorectal cancer. *Br J Cancer* **70**: 1150–1155

Fidanza F and Verdiglioni M (1988) Tabelle di composizione degli alimenti. In *Nutrizione Umana*, Fidanza F, Liguori G (eds), pp. 677–730. Idelson: Naples

Franceschi S, La Vecchia C, Bidoli E, Negri E and Talamini R (1992) Meal frequency and risk of colorectal cancer. *Cancer Res* **52**: 3589–3592

Fuchs CS, Giovannucci EL, Colditz GA, Hunter DJ, Speizer FE and Willett WC (1994) A prospective study of family history and the risk of colorectal cancer. *N Engl J Med* **331**: 1669–1674

Kelly JP, Rosenberg L, Kaufman DW and Shapiro S (1990) Reliability of personal interview data in a hospital-based case–control study. *Am J Epidemiol* **131**: 79–90

Kune GA, Kune S and Watson LF (1989) The role of heredity in the etiology of large bowel cancer. *World J Surg* **13**: 124–129

La Vecchia C, Negri E, Decarli A, D'Avanzo B, Galloti L, Gentile A and Franceschi S (1988) A case–control study of diet and colo-rectal cancer in Northern Italy. *Int J Cancer* **41**: 492–498

La Vecchia C, Negri E, Franceschi S and Gentile A (1992) Family history and the risk of stomach and colorectal cancer. *Cancer* **70**: 50–55

La Vecchia C, Ferraroni M, Mezzetti M, Enard L, Negri E, Franceschi S and Decarli A (1996) Attributable risks for colorectal cancer in Northern Italy. *Int J Cancer* **66**: 60–64

Levi F, Lucchini F and La Vecchia C (1994) Worldwide patterns of cancer mortality, 1985–89. *Eur J Cancer Prev* **3**: 109–143

Ponz De Leon M, Sassatelli R, Sacchetti C, Zanghieri G, Scalmati A and Roncucci L (1989) Familial aggregation of tumors in the three-year experience of a population-based colorectal cancer registry. *Cancer Res* **49**: 4344–4348

Potter JD, Slattery ML, Bostick RM and Gapstur SM (1993) Colon cancer: a review of the epidemiology. *Epidemiol Rev* **15**: 499–545

Slattery ML and Kerber RA (1994) Family history of cancer and colon cancer risk, the Utah population database. *J Natl Cancer Inst* **86**: 1618–1626

Slattery ML, Mineau GP and Kerber RA (1995) Reproductive factors and colon cancer: the influences of age, tumor site, and family history on risk (Utah, United States). *Cancer Causes Control* **6**: 332–338

St John DJ, McDermott FT, Hopper JL, Debney EA, Johnson WR and Hughes ES (1993) Cancer risk in relatives of patients with common colorectal cancers. *Ann Intern Med* **118**: 785–790

Willett WC (1989) The search for the causes of breast and colon cancer. *Nature* **338**: 389–394

 © Cancer Research Campaign 1997